# Characterization of Magnetic Biochar Modified Using the One-Step and Electrochemical Methods and Its Impact on Phosphate Adsorption

**DOI:** 10.3390/ma16227092

**Published:** 2023-11-09

**Authors:** Changgen Mei, Lulu Wang, Wei Tao

**Affiliations:** School of Chemistry and Environmental Engineering, Sichuan University of Science and Engineering, Zigong 643000, China

**Keywords:** one-step method, electrochemical method, Fe/Mg ratio, magnetic biochar, phosphate adsorption

## Abstract

The properties and phosphate adsorption capability of the one-step method and electrochemical method in modifying peanut shell biochar have been determined. The one-step method deposits MgO and Fe_3_O_4_ onto biochar through chemical impregnation and regularly affects the functional groups and magnetic separation of biochar, thereby enhancing its ability to adsorb phosphate. In contrast, the electrochemical method is not favorable for modifying functional groups of biochar but can promote phosphate adsorption because of the formation of MgFe_2_O_4_ and Fe_3_O_4_ using electrolysis. The adsorption isotherm and kinetics data suggest that adsorption is monolayer onto a homogeneous surface and phosphate adsorption could be controlled by chemical processes. Biochar with the addition of both Fe^2+^ and Mg^2+^ shows better phosphate adsorption capability than those with barely any Fe^2+^ additions. It was concluded that the one-step method is a better modification method than the electrochemical method for enhancing the phosphate adsorption capability of biochars.

## 1. Introduction

Phosphate-contaminated waters not only increase the cost of wastewater treatment but can also lead to the formation of harmful algae that may produce cyanotoxins and threaten human health [1,2], so the treatment of phosphates has received increasing attention. Biochar is obtained from the pyrolytic conversion of biomass or pretreated biomass through the maintenance of an oxygen-free or other atmosphere [3,4]. Its properties include a large surface area, high porosity, and a stable carbon matrix [5]. However, the separation of the biochar and liquid solution has limited the application of biochar in adsorbing common anionic pollutants in wastewater. Therefore, the development of magnetic biochar offers the opportunity to address the difficulties faced in the application of biochar in wastewater. Numerous studies have been conducted on modification methods. Some studies modified biochar with the one-step method, in which the raw material is impregnated with a metal solution and, subsequently, pyrolyzed at a different temperature [6,7,8,9,10,11]. The obtained magnetic biochars comprised nano-sized Fe_x_O_y_/MgO particles and exhibited a great affinity for phosphate in a liquid solution [6]. Because the point of the zero charge (pH_pzc_) of MgO (approximately 12) is higher than that of Fe_3_O_4_ (approximately 7), a few studies have found that an increase in the Mg^2+^ ratio changed the surface charge of biochar, thus promoting the efficiency of magnesium-decorated magnetic biochars in adsorbing phosphate [12]. Under this mechanism, the addition of a different Fe^2+^/Mg^2+^ ratio during the synthesis process might have a different effect on the surface charge of magnetic biochar and, thus, impose different effects on the adsorption ability of magnetic biochar. Some studies modified biochar with the chemical coprecipitation technique [13], in which the obtained magnetic biochar showed a strong adsorption capacity for anionic pollutants. Furthermore, to shorten the biochar modification time, some studies [14,15] modified biochar with an electrolytic electrolyte solution using electrodes, which changed the physical–chemical properties of biochar and can greatly improve phosphate adsorption [14,15]. Modification using the electrochemical and one-step methods can change the adsorption capacity of biochar to varying degrees. However, the differences between the modification methods, such as the mechanism of modification, the microscopic differences of the modified biochar, the effect of Mg^2+^ addition, and the mode of adsorption, have yet to be investigated. Therefore, it is necessary to explore the differences between the electrochemical and one-step methods.

Therefore, the aim of this study is to prepare Fe/Mg composite biochar from peanut shell biomass with the electrochemical and one-step methods, make comparisons between the different aspects, such as surface properties, microstructure, adsorption mode, etc., and ultimately, evaluate the differences between the two modification methods.

## 2. Materials and Methods

### 2.1. Material and Preparation of Raw Biochar

All chemicals, including NaOH, HCl, H_2_SO_4_, KH_2_PO_4_, MgCl_2_, and FeCl_2_, used in this study were purchased in analytical grade from Chron Chemicals (Chengdu, China) and used as received.

This work used agricultural waste in the form of peanut shells as the raw material for synthesizing biochar. The peanut shells were collected from the agricultural region of Yichun City, Jiangsu Province (China). The peanut shells were washed three times with deionized (DI) water to remove impurities and then dried in an oven at 60 °C for 24 h. Thereafter, the dried samples were ground into powder and then sieved to a particle size of 30 to 60 meshes. The ground peanut shells were then pyrolyzed in a furnace (SK3-2-10-8) under N2 conditions (140 mL/min) at 600 °C (50 °C/min) for 1 h, and then the pyrolyzed sample was cooled to room temperature in an N_2_ environment. Subsequently, the collected sample was ground and sieved through 60 to 100 meshes; the meshed sample was washed with 0.05 M NaOH/HCl for 24 h to remove the soluble matter and then rinsed with DI water until the electrical conductivity of water was less than 5 μS/cm. Finally, raw biochar was obtained by drying the sample at 80 °C for 12 h and labeled as BC.

### 2.2. Preparation of Magnetic Biochar

In this study, magnetic biochar was prepared using two methods, namely, the one-step and electrochemical methods.

In the one-step method, 400 mL 0.04 M FeCl_2_·4H_2_O was mixed with MgCl_2_·6H_2_O in a concentration of 0 M, 0.04 M, and 0.08 M, respectively. Then, 20 g of ground peanut shells were added to these solutions to form a mixture with a solid–liquid ratio of 1:20. After adjusting the pH of the mixture to 11 ± 0.03, it was sealed with plastic wrap, left to stand for 8 h, and then filtrated to harvest the solids. The harvested solids were dried in an oven (80 °C, 12 h) and then pyrolyzed by following the pyrolysis protocol of raw biochar. Finally, the sample was ground and sieved through 60 to 100 meshes, rinsed with DI water, and dried in an oven (80 °C, 12 h). The acquired magnetic biochar was expressed as A-BC, B-BC, and C-BC, where A, B, and C indicate that the mole ratios of Fe^2+^ to Mg^2+^ are 1:0, 1:1, and 1:2, respectively.

In the electrochemical method, mixtures of peanut shell, FeCl_2_·4H_2_O, and MgCl_2_·6H_2_O were prepared following the protocol described in the one-step method. Afterward, the pH of the mixture was adjusted to 3 ± 0.03, and then a current with a density of 2 A was applied to the mixture for 30 min under constant stirring, in which graphite was used as an anode and cathode and the effective electrolysis area was 45.65 cm^2^. Then, the solids were harvested using filtration and dried in an oven (80 °C, 12 h) and then pyrolyzed following the pyrolysis protocol of raw biochar. Finally, the sample was ground and sieved through 60 to 100 meshes, washed with DI water, and dried in an oven (80 °C, 12 h). The acquired magnetic biochar was expressed as D-BC, E-BC, and F-BC, where D, E, and F mean the mole ratios of Fe^2+^ to Mg^2+^ are 1:0, 1:1, and 1:2, respectively.

### 2.3. Characterizations of Biochar

To quantify the functional groups of the biochars, the Fourier transform infrared spectrometry (FTIR) data were recorded in 4000~500 cm^−1^ (4 cm^−1^ resolution, 32 scanning frequency) using a NICOLET 6700 spectrophotometer (Thermo Fisher Scientific, Waltham, MA, USA). Meanwhile, the crystal structures of the biochars were measured by a D2-PHASER (Bruker AXE, Karlsruhe, Germany). The magnetism of the magnetic biochar was measured using the vibrating sample magnetometer (VSM; Lake Shore Cryotroni, Westerville, OH, USA) at room temperature. Moreover, the point of zero charge (pH_pzc_) was obtained using the method proposed by Liu et al. [16] and Tao et al. [12]. Briefly, 0.1 g of the biochar samples was mixed with 50 mL of 0.01 M NaNO_3_ in a 100 mL beaker. Then, the pH (pH_i_) of the mixture was adjusted using 1 M NaOH and 1 M HNO_3_, ranging from 4 to 10. Thereafter, the beaker was sealed with plastic wrap and shaken for 24 h (120 rpm, 30 °C), and the pH (pH_f_) of the mixture was measured using a pH electrode (METTLER TOLEDO, Suzhou, China). Then, the pH_pzc_ value of biochar was obtained from the graph of (pH_i_-pH_f_) against pH_i_. Furthermore, acidic and basic oxygen-containing functional groups (both acidic and basic) of biochar were quantified using the Boehm titration method [17,18,19]. Finally, the change in the pH of biochar with time was also tested with a pH electrode (METTLER TOLEDO, Suzhou, China). Specifically, 0.5 g of the biochar sample was added to 100 mL of DI water, the pH change in the biochar mixed with the aqueous solution was measured within 2 h, and the data were recorded every minute for the first 4 min and every 5 min, thereafter.

### 2.4. Phosphate Adsorption Studies

In the batch sorption experiments, 50 mg/L of a K_2_HPO_4_ solution was prepared as the stock solution. A total of 0.1 g of each biochar sample was mixed with 50 mL of the phosphate stock solution in a 250 mL beaker. The beakers were sealed with plastic wrap and shaken in a gas bath thermostatic oscillator (120 rpm and 25 °C), and samples were taken at preset times. In the isothermal adsorption experiments, 0.1 g of each biochar sample was mixed with 50 mL of phosphate solution, with a concentration ranging from 5 to 50 mg·L^−1^ in a 250 mL beaker. The beakers were sealed with plastic wrap and shaken for 24 h (120 rpm and 25 °C). Afterward, all mixtures were filtered through a Whatman filter (0.45 μm), and the phosphate concentration in the filtrate was determined using spectrophotometry at a wavelength of 700 nm. In this study, batch experiments were carried out in triplicate, and the phosphate adsorption capability of biochar was calculated as follows:

(1) $$Q_{t}=V(C_{1}-C_{0})/M$$

where *Q_t_* is the amount of phosphate adsorbed per unit mass of biochar at a given time (mg/g), *V* is the volume of phosphate (L), *C*_0_ and *C*_1_ are the concentration of phosphate before and after adsorption (mg/L), respectively, and *M* is the mass of biochar (g).

### 2.5. Data Analysis

Kinetic data were fitted through three kinetic models: pseudo-first-order, pseudo-second-order, and intra-particle diffusion, using equations:

(2) $$q_{t}=q_{e}(1-e^{-k_{1}t})$$

(3) $$q_{t}=\frac{q_{e}^{2}k_{2}t}{1+q_{e}k_{2}t}$$

(4) $$q_{t}=k_{i}t^{1/2}+C$$

where *t* is the adsorption time, *q_t_* is the amount of phosphate adsorbed onto biochars at time *t*, *q_e_* is the amount of phosphate adsorbed at equilibrium, *k*_1_, *k*_2_, and *k_i_* are the rate constants of pseudo-first, pseudo-second, and intra-particle diffusion, and C is a constant describing the thickness of the boundary layer.

Three isotherm models, namely, Langmuir, Freundlich, and Temkin [8,20], were employed to study the equilibrium concentration data obtained from the adsorption of phosphate onto the biochars; the equations are as follows:

(5) $$q_{e}=\frac{q_{max}K_{L}C_{e}}{1+K_{L}C_{e}}$$

(6) $$q_{e}=K_{F}C_{e}^{1/n}$$

(7) $$q_{e}=\frac{RT}{\beta_{T}}ln\left(A_{T}C_{e}\right)$$

where *q_e_* is the equilibrium adsorption capacity(mg/g), *q_max_* is the maximum adsorption capacity (mg/g), *C_e_* is the equilibrium concentration of adsorbate (mg/L), *K_L_* is the Langmuir isotherm constant (L/mg), *K_F_* is the Freundlich isotherm constant (mg/g(L/mg)1/n), *β_T_* is the Temkin constant related to the heat of sorption (J/mol), *A_T_* is the Temkin isotherm constant (L/mg), *R* is the gas constant (8.314 J/mol·K), and *T* is the absolute temperature (K).

## 3. Results and Discussion

### 3.1. Biochar Characterizations

#### 3.1.1. FTIR and XRD Analysis

**Figure 1:**
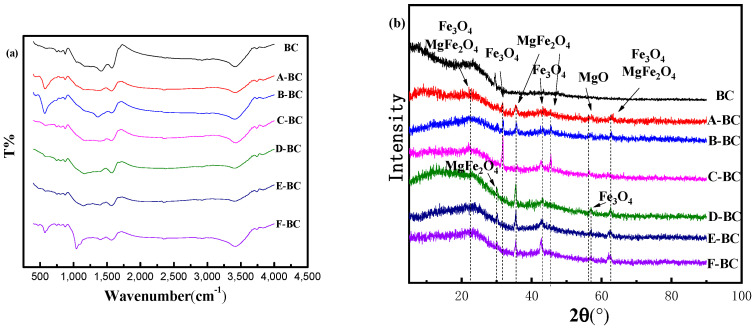
(**a**) FTIR spectrum and (**b**) XRD patterns of biochar.

To determine the functionalities of biochar, FTIR spectra were performed within a range of 4000 to 500 cm^−1^, as shown in Figure 1a. For instance, the appearance of a peak at approximately 3400 cm^−1^ may be attributed to –OH functional groups or alcohols and aldehydes [21]. The peak appearing at approximately 2972 cm^−1^ might be attributed to the antisymmetric stretching peak of –CH_3_, the peak appearing at 2346 cm^−1^ (2300–2400 cm^−1^) might be attributed to –COOH and C=O, the peak appearing at 1572 cm^−1^ may be attributed to a C=C stretching vibration caused by the aromatic structure [16], the peak observed at approximately 1411 cm^−1^ might be attributed to the scissor bending vibration of –CH_2_, and the peak near 873 cm^−1^ is characteristic of C–H bending vibration in a b-glucosidic linkage [16]. Moreover, the peak ranging from 700 to 400 cm was attributed to the stretching vibration of Fe-O [22], as well as the peak at approximately 568 nm in all magnetic biochars, rather than the biochar reflecting the introduction of iron oxide into magnetic biochars. The peak intensity decreased with the decrease in the Fe-Mg ratio, which might have occurred because an increase in magnesium will reduce the formation of iron–oxygen bonds. Furthermore, the difference between magnetic biochars generated using the one-step and electrochemical methods is that a stretching vibration appears at 1165 cm^−1^ on the FTIR spectrum of the magnetic biochars prepared using the electrochemical method. The stretching vibration appearing at 1165 cm^−1^ could be attributed to a C-O stretching vibration peak [21] or a C-N stretching vibration peak.

The XRD patterns of biochars were measured to study their crystal structure, as shown in Figure 1b. The peak located at the 2θ angles of 22.1°, 30.4°, 42.6°, 58.1°, and 62.5° suggested the occurrence of Fe_3_O_4_ (JCPDS 19-629) [23], revealing that the magnetic iron oxide was loaded on the biochar successfully. The intensity of these peaks decreasing with the increase in the Mg^2+^ ratio reveals that the magnetic properties also decreased with the increase in the Mg^2+^ ratio. Meanwhile, the peak of MgFe_2_O_4_ with a cubic spinel structure located at the 2θ angles of 22.1°, 29.8°, 35.1°, 45.4°, and 62.2° also increased the magnetic properties of biochar to some extent (JCPDS 73–2410) [24,25]. In addition, the peak of magnesium oxide (MgO) appeared at 42.5° (JCPDS 45–0946) [26]. Comparing the XRD patterns of magnetic biochar produced using the one-step (chemical impregnation) and electrochemical modification methods, the peak of MgO only appears in the XRD patterns of the magnetic biochar produced using the one-step method (chemical impregnation). However, the peaks of MgFe_2_O_4_ located at 29.8° and Fe_3_O_4_ located at 58.1° in the XRD patterns of magnetic biochar produced using the electrochemical method are not found in the biochar produced using the one-step method (chemical impregnation). The difference between these two methods indicates that the electrochemical method promotes the conversion of Mg and Fe to MgFe_2_O_4_ substances.

#### 3.1.2. Hysteresis Loops

**Figure 2:**
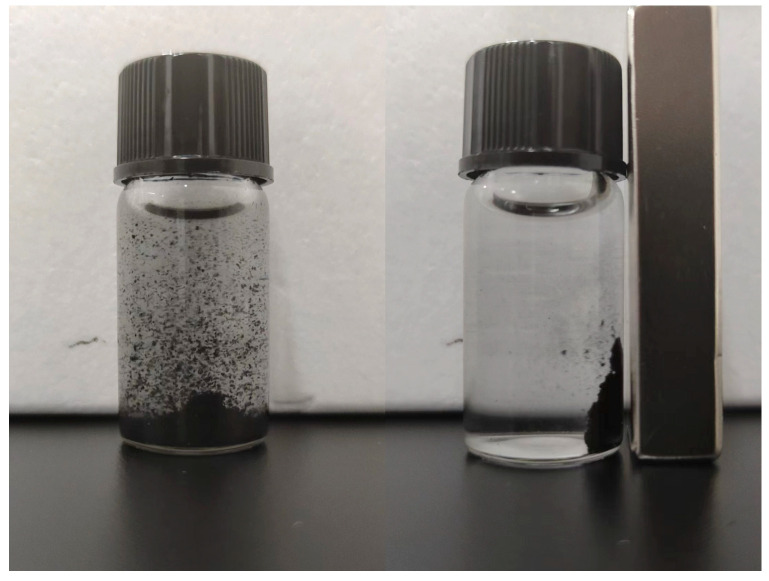
Magnetic separation of A-BC from liquid solution.

The magnetic separation of A-BC from the liquid solution shown in Figure 2 holds true for other biochars, implying the possibility of separating biochars after adsorption. After rotating the mixture of biochars and DI water at room temperature at 120 rpm for 7 days, the magnetic biochars were still easily separated using a magnet, indicating that the magnetic biochar is stable in solutions.

**Figure 3:**
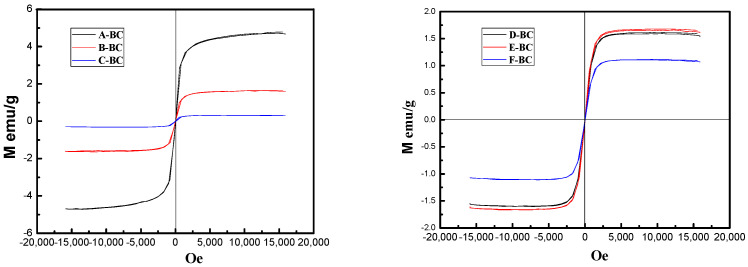
Magnetic hysteresis curves of biochars.

To further understand the magnetism of different magnetic biochars, the magnetic field-dependent behaviors were measured, as shown in Figure 3. Normal narrow hysteresis loops (S-shaped) were obtained for all magnetic biochars, and the remanence and coercivity were almost equal to zero, which is a typical characteristic of spinel ferrite materials [27]. These results indicate that the magnetic biochars produced with both the one-step method and the electrochemical method have superparamagnetic properties [28].

As shown in Figure 3, the magnetic properties of the biochars prepared using the one-step method gradually decreased with the increase in the molar ratio of Mg^2+^ ions from 1:0 to 1:2, which were 4.66 emu/g, 1.61 emu/g, and 0.29 emu/g, respectively. This phenomenon is due to the gradual increase in Mg-containing compounds attached to the peanut shell biomass as the molar ratio of Mg^2+^ increases during the one-step process and the replacement of some of the sites of Fe substances by Mg-containing substances during the soaking process. In addition, some of the metal ions present in the solution are lost during filtration, leading to a decrease in magnetic iron oxide production after pyrolysis, which corresponds to the FTIR test results; as a consequence, the magnetic properties of the biochar are reduced.

However, the magnetic difference among biochars modified using the electrochemical method is not significantly affected by the molar ratio of Mg^2+^. This phenomenon occurs due to the electrolysis of iron-containing electrolytes, during which part of the iron-containing material exists in the water and is eventually lost during filtration, whereas only a fraction of the iron-containing material is converted into magnetic iron oxide. In addition, the electrolysis process depends on the strength of the electrolysis, such as the current, the effective area of the electrode, the concentration of the solution, etc. In this study, the strength of the electrical stimulation was limited, and the electrolysis could only reach a certain threshold because the electrolysis process was performed on a limited electrolysis area with 2A of electrolysis current for 30 min.

Thus, it can be concluded that the one-step method can deposit iron and magnesium substances onto peanut shells regularly with different ratios using chemical impregnation and transform them into oxides favorable for adsorption. However, the electrochemical method only relies on electrolytic strength to utilize Fe^2+^ and Mg^2+^ in the solution under the action of the electric field and can promote the production of MgFe_2_O_4_ in the spinel structure, thus increasing its magnetic separation effect.

#### 3.1.3. pH_pzc_ and pH

pH_pzc_ was used to understand the surface charging of biochar solids (Table 1) because pH_pzc_ plays an important role in identifying electrostatic attraction between the adsorbent and the adsorbate [13,29]. The pH_pzc_ of all magnetic biochar composites is greater than in unmodified biochar, which is consistent with the findings of Tao et al. [12], that the introduction of Fe and Mg oxides on biochar increased the pH_pzc_ of all magnetic biochar composites. Moreover, the pH_pzc_ of all Fe/Mg magnetic biochar composites is greater than pH_pzc_ > 8 because the pHpzc of MgO (approximately 12) [30] is greater than Fe_3_O_4_ (approximately 7) [31]. These results imply that all Fe/Mg magnetic biochar is positively charged at a pH lower than their pH_pzc_ and negatively charged at a pH greater than their pH_pzc_. These results also imply that the introduction of Fe and Mg oxides to biochar provides an opportunity for the electrostatic adsorption of negatively charged anions [8].

**Figure 4:**
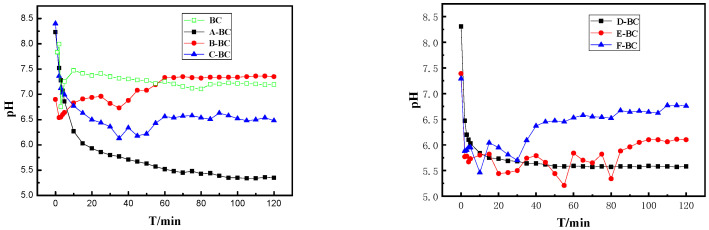
pH of magnetic biochar in suspension.

Figure 4 shows the change in pH of magnetic biochar in suspension. With the increase in time in an aqueous solution, the pH value of a biochar sample first fluctuates several times, and then gradually stabilizes. The pH of B-BC has the smallest fluctuation range compared to the rest of the magnetic biochar, with a final pH of 7, indicating a smaller effect on the pH of the solution. The changes in pH might be attributed to the different functional groups of magnetic biochar prepared using different methods because the pH of biochar depends on its types of functional groups [32].

#### 3.1.4. Boehm Titration

**Figure 5:**
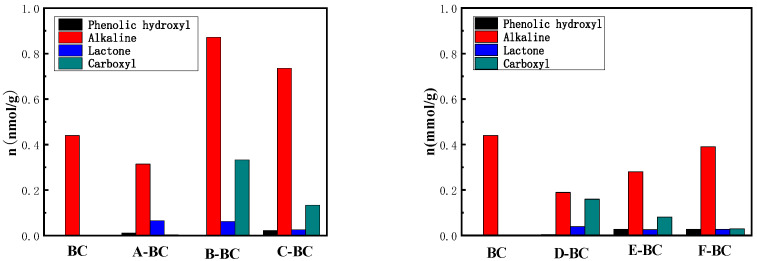
Boehm titration of biochar.

To further study the functional groups of prepared biochar, Boehm titration was performed. As shown in Figure 5, the number of functional groups (phenolic hydroxyl, lactone, carboxyl, and alkaline) on the magnetic biochars prepared using the two modification methods varied greatly. Specifically, the one-step method increased the number of alkaline groups on the biochar samples based on the modification of alkaline-oxidized metals, and thus, increased the alkalinity of the biochar, which is consistent with the result of pH_pzc_. Moreover, with the increase in the molar ratio of Mg^2+^ ions, the number of lactone groups decreased, whereas the carboxyl group and a small number of phenolic hydroxyl groups on the biochar increased. These changes are conducive to the adsorption of biochar. For magnetic biochars produced using the electrochemical modification method, the number of alkaline groups was less than that of BC, but increased with the increase of the Mg^2+^ molar ratio, whereas the number of carboxyl groups decreased with the increase in the Mg^2+^ molar ratio. These results reveal that the electrochemical method is not favorable for the modification of functional groups.

### 3.2. Adsorption of Phosphate

Unmodified biochar has a negative adsorption capacity for phosphate (−0.056 mg/g), that is, in the phosphate solution, the content of phosphorus released by biochar is greater than the amount of adsorption; as a consequence, the adsorption of unmodified biochar is not discussed in the following section.

#### 3.2.1. Adsorption Kinetics

**Figure 6:**
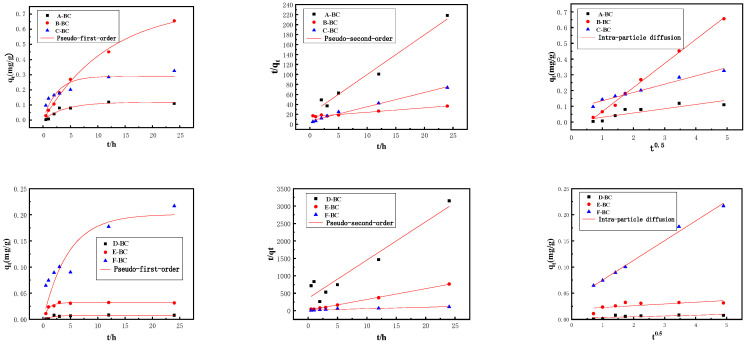
Adsorption kinetics of phosphate adsorption onto magnetic biochar.

The adsorbed amount of phosphate per gram of magnetic biochar as a function of time is shown in Figure 6. All magnetic biochars share similar adsorption trends as time passes, specifically, after very rapid initial adsorption, the adsorption rate increases slowly and reaches equilibrium. To understand the adsorption behavior of phosphate, all data were fitted using kinetic models, including the pseudo-first-order model, pseudo-second-order model, and intra-particle diffusion model, and kinetic parameters are listed in Table 2. The pseudo-second-order model fitted the data somewhat better than the pseudo-first-order and intra-particle diffusion models, with all correlation coefficients (*R*^2^) exceeding 0.9060 (Table 2). This result is consistent with the previous finding that phosphate adsorption on magnetic biochars was mainly controlled using chemisorption [33]. For magnetic biochars produced using the two methods, an increase in the Mg^2+^ molar ratio increased phosphate adsorption.

The modification method of magnetic biochar also affects the phosphate adsorption process. For magnetic biochar produced using the one-step method, the pseudo-first-order model and intra-particle diffusion model also agreed with the experiment data. These results indicated that the pore structure and volume of magnetic biochar also play an important role in its adsorption capacity. However, for magnetic biochar produced using the electrochemical method, the pseudo-second-order model also shows better fitting than the other two models, indicating that other factors did not significantly affect phosphate adsorption, besides the chemical sorption. These differences between the two types of magnetic biochar suggest that one-step-modified biochar can promote adsorption in all aspects, while electrochemical-modified biochar can only improve its adsorption capacity with the chemical adsorption of ferromagnesium oxide composites.

#### 3.2.2. Adsorption Isotherm

**Figure 7:**
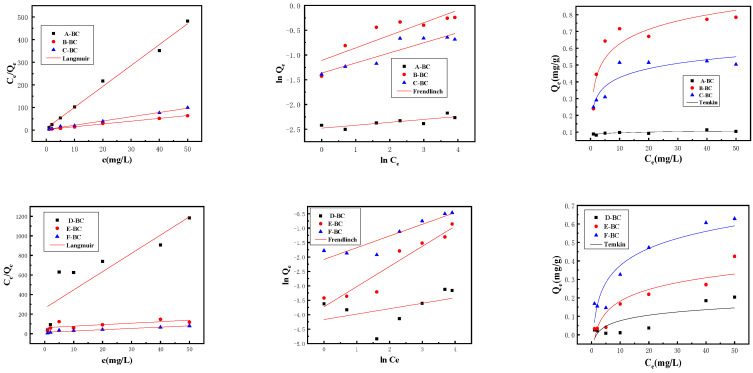
Adsorption thermodynamics of phosphate onto magnetic biochar.

To further understand the adsorption mechanism, the equilibrium concentration was fitted using the Langmuir, Freundlich, and Temkin models, as shown in Figure 7, and the fitted parameters are listed in Table 3.

The fitting of Langmuir matches the adsorption data of magnetic biochar, suggesting that adsorption is monolayer adsorption onto a homogeneous surface, and there is no interaction between adsorbents [8]. However, for E-BC, the fitting of the Freundlich model matches the experimental data better than the Langmuir model, indicating the existence of inhomogeneous surface adsorption. The Langmuir maximum capacity of B-BC is approximately 0.8083 mg/g, which is lower than what has been reported in previous work [7,8,12], in which different raw materials were employed to synthesize biochar. A possible reason might be the biochar produced using peanut shells is not favorable for phosphate adsorption. In addition, the Freundlich constant (1/n) that represents the heterogeneity of the site energy is classified into five categories in the study of Tseng and Wu [34], in which the smaller 1/n, the more conducive to adsorption. In all cases, 1/n values are less than 1, indicating that the adsorption of phosphate onto biochars is favorable [35,36]. For biochars prepared using the one-step method, the 1/n value was reduced with the increase in the Mg^2+^ ratio, indicating the increase in the adsorption site number or the composite strength, which is conducive to adsorption [37]. In contrast, in biochars prepared using the electrochemical method, the 1/n value increased with the increase in the Mg^2+^ ratio, which indicates that the electrochemical method decreased the number of adsorption sites. In addition, the *K_F_* value increased with the increase in the Mg^2+^ ratio. According to XRD analysis, the modified Fe/Mg oxide is conducive to enhancing the phosphate sorption capacity of magnetic biochar.

## 4. Conclusions

In summary, the magnetic biochars prepared using the one-step and electrochemical methods were compared, and their phosphate adsorption capability was evaluated. The one-step method successfully loaded MgO and Fe_3_O_4_ onto biochars, and the electrochemical method loaded MgFe_2_O_4_ and Fe_3_O_4_ onto biochars; as a consequence, all the biochars have magnetic separation characteristics. In addition, the introduction of Fe and Mg oxides increased the pH_pzc_ of the biochars, which provided an opportunity for the electrostatic adsorption of negatively charged anions. Phosphate adsorbed onto magnetic biochar produced using the one-step and electrochemical methods is mainly controlled using chemical adsorption. The maximum phosphate adsorption capacity was as high as 0.8083 mg/g, as fitted using the Langmuir model. The undesirable phosphate adsorption capacity might be attributed to the fact that peanut shell biochar is not favorable for phosphate adsorption. Biochar prepared in the presence of both Fe^2+^ and Mg^2+^ shows better adsorption capability for phosphate than biochars with barely any Fe^2+^ additions, while the increase in the Mg^2+^ ratio did not show a significant impact on phosphate removal. On the whole, biochars modified using the one-step method show a somewhat better phosphate adsorption capability than biochars modified using the electrochemical method.

## Figures and Tables

**Table 1:** The pHpzc of biochars in suspension.

Materials	BC	A-BC	B-BC	C-BC	D-BC	E-BC	F-BC
pH_pzc_	5.45	6.49	9.08	8.81	4.52	8.71	9.90

**Table 2:** Kinetic parameters of phosphate adsorption onto magnetic biochar.

Kinetic Model	Parameters	A-BC	B-BC	C-BC	D-BC	E-BC	F-BC
Pseudo-First-Order	*q_e_* (mg/g)	0.1174	0.7502	0.2901	0.0079	0.0318	0.2009
	*K*_1_ (1/h)	0.2487	0.0831	0.3946	0.4933	1.0729	0.0218
	*R* ^2^	0.9125	0.9958	0.7295	0.7419	0.9258	0.6528
Pseudo-Second-Order	*q_e_* (mg/g)	0.1257	1.1305	0.3495	0.0091	0.0323	0.2411
	*K*_2_ [g/(mg·h)]	3.1028	0.0509	1.2387	33.9153	81.0668	1.0928
	*R* ^2^	0.9695	0.9688	0.9576	0.9060	0.9980	0.9202
Intra-Particle Diffusion	*K* [g/(mg·h^1/2^)]	0.0271	0.1529	0.0527	0.0018	0.0036	0.0376
	*C*	0.0031	0.0869	0.0825	0.0215	0.0194	0.0332
	*R* ^2^	0.7045	0.9966	0.9616	0.6970	0.6498	0.9751

**Table 3:** Adsorption isotherm parameters of phosphate onto magnetic biochar.

Isotherm Model	Parameters	A-BC	B-BC	C-BC	D-BC	E-BC	F-BC
Langmuir	*q_max_* (mg/g)	0.3093	0.8083	0.5307	0.0535	0.6586	0.7620
	*K_L_* (L/mg)	0.3729	0.4975	0.6683	0.0718	0.0245	0.0885
	*R* ^2^	0.9912	0.9968	0.9953	0.7318	0.3947	0.9143
Freundlich	*K_F_* (L/mg)	0.0834	0.3292	0.2554	0.0155	0.0241	0.1246
	1/n	0.5883	0.2536	0.2037	0.1880	0.6986	0.4060
	*R* ^2^	0.6023	0.7316	0.8217	0.0663	0.8922	0.8284
Temkin	*β_T_* (J/mol)	2.4179	15.4922	25.1650	0.5818	0.7342	1.6556
	*A_T_* (L/mg)	0.0056	0.1241	0.0769	0.0432	0.0913	0.1336
	*R* ^2^	0.5901	0.8321	0.8030	0.4794	0.8060	0.8452

## Data Availability

Data are contained within the article.

## References

[1] Lalley J., Han C., Li X., Dionysiou D.D., Nadagouda M.N. (2016). Phosphate adsorption using modified iron oxide-based sorbents in lake water: Kinetics, equilibrium, and column tests. Chem. Eng. J..

[2] Wu J., Wang J., Du Y., Li H., Jia X. (2016). Adsorption mechanism and kinetics of azo dye chemicals on oxide nanotubes: A case study using porous CeO_2_ nanotubes. J. Nanopart. Res..

[3] Ahmad M., Rajapaksha A.U., Lim J.E., Zhang M., Bolan N., Mohan D., Vithanage M., Lee S.S., Ok Y.S. (2014). Biochar as a sorbent for contaminant management in soil and water: A review. Chemosphere.

[4] Xiao R., Sun X., Wang J., Feng J., Li R., Zhang Z., Wang J.J., Amjad A. (2015). Characteristics and phytotoxicity assay of biochars derived from a Zn-rich antibiotic residue. J. Anal. Appl. Pyrolysis.

[5] Li R., Wang J.J., Gaston L.A., Zhou B., Li M., Xiao R., Wang Q., Zhang Z., Huang H., Liang W. (2018). An overview of carbothermal synthesis of metal–biochar composites for the removal of oxyanion contaminants from aqueous solution. Carbon.

[6] Chen B., Chen Z., Lv S. (2011). A novel magnetic biochar efficiently sorbs organic pollutants and phosphate. Bioresour. Technol..

[7] Zhang M., Gao B., Yao Y., Xue Y., Inyang M. (2012). Synthesis of porous MgO-biochar nanocomposites for removal of phosphate and nitrate from aqueous solutions. Chem. Eng. J..

[8] Yao Y., Gao B., Inyang M., Zimmerman A.R., Cao X., Pullammanappallil P., Yang L. (2011). Removal of phosphate from aqueous solution by biochar derived from anaerobically digested sugar beet tailings. J. Hazard. Mater..

[9] Fang C., Zhang T., Li P., Jiang R., Wu S., Nie H., Wang Y. (2015). Phosphorus recovery from biogas fermentation liquid by Ca–Mg loaded biochar. J. Environ. Sci..

[10] Li R., Wang J.J., Zhou B., Awasthi M.K., Ali A., Zhang Z., Gaston L.A., Lahori A.H., Mahar A. (2016). Enhancing phosphate adsorption by Mg/Al layered double hydroxide functionalized biochar with different Mg/Al ratios. Sci. Total Environ..

[11] Zhang C., Dong Y., Yang D., Jin Q., Lin H. (2023). Synthesis of co-pyrolyzed biochar using red mud and peanut shell for removing phosphate from pickling wastewater: Performance and mechanism. Chemosphere.

[12] Tao X., Huang T., Lv B. (2020). Synthesis of Fe/Mg-Biochar Nanocomposites for Phosphate Removal. Materials.

[13] Reguyal F., Sarmah A.K., Gao W. (2017). Synthesis of magnetic biochar from pine sawdust via oxidative hydrolysis of FeCl2 for the removal sulfamethoxazole from aqueous solution. J. Hazard. Mater..

[14] Jung K.-W., Choi B.H., Song K.G., Choi J.-W. (2019). Statistical optimization of preparing marine macroalgae derived activated carbon/iron oxide magnetic composites for sequestering acetylsalicylic acid from aqueous media using response surface methodologys. Chemosphere.

[15] Zoroufchi Benis K., Sokhansanj A., Norberto J., McPhedran K.N., Soltan J. (2022). A binary oxide-biochar composite for adsorption of arsenic from aqueous solutions: Combined microwave pyrolysis and electrochemical modification. Chem. Eng. J..

[16] Liu Y., Zhao X., Li J., Ma D., Han R. (2012). Characterization of bio-char from pyrolysis of wheat straw and its evaluation on methylene blue adsorption. Desalination Water Treat..

[17] Choudhary M., Kumar R., Neogi S. (2020). Activated biochar derived from Opuntia ficus-indica for the efficient adsorption of malachite green dye, Cu^+2^ and Ni^+2^ from water. J. Hazard. Mater..

[18] Boehm H.P., Eley D.D., Pines H., Weisz P.B. (1966). Chemical Identification of Surface Groups. Advances in Catalysis.

[19] Schönherr J., Buchheim J.R., Scholz P., Adelhelm P. (2018). Boehm Titration Revisited (Part I): Practical Aspects for Achieving a High Precision in Quantifying Oxygen-Containing Surface Groups on Carbon Materials. C.

[20] Fang C., Zhang T., Li P., Jiang R.-f., Wang Y. (2014). Application of Magnesium Modified Corn Biochar for Phosphorus Removal and Recovery from Swine Wastewater. Int. J. Environ. Res. Public Health.

[21] Chen B., Zhou D., Zhu L. (2008). Transitional Adsorption and Partition of Non-Polar and Polar Aromatic Contaminants by Biochars of Pine Needles with Different Pyrolytic Temperatures. Environ. Sci. Technol..

[22] Gong C., Chen D., Jiao X., Wang Q. (2002). Continuous hollow alpha-Fe_2_O_3_ and alpha-Fe fibers prepared by the sol-gel method. J. Mater. Chem..

[23] Aryee A.A., Dovi E., Han R., Li Z., Qu L. (2021). One novel composite based on functionalized magnetic peanut husk as adsorbent for efficient sequestration of phosphate and Congo red from solution: Characterization, equilibrium, kinetic and mechanism studies. J. Colloid Interface Sci..

[24] Ghasemzadeh M.A., Mirhosseini-Eshkevari B., Sanaei-Rad S. (2022). ZIF-8-incorporated nanoparticles of MgFe_2_O_4_ supported on graphene oxide: A ternary hybrid catalyst for the efficient synthesis of pyrazole-based pyrido[2,3-d]pyrimidine-diones. Polyhedron.

[25] Huynh N.C., Nguyen T.T.T., Nguyen D.T.C., Tran T.V. (2023). Production of MgFe_2_O_4_/activated carbons derived from a harmful grass Cynodon dactylon and their utilization for ciprofloxacin removal. Chemosphere.

[26] Wu L., Wei C., Zhang S., Wang Y., Kuzyakov Y., Ding X. (2019). MgO-modified biochar increases phosphate retention and rice yields in saline-alkaline soil. J. Clean. Prod..

[27] Jung K.-W., Lee S., Lee Y.J. (2017). Synthesis of novel magnesium ferrite (MgFe_2_O_4_)/biochar magnetic composites and its adsorption behavior for phosphate in aqueous solutions. Bioresour. Technol..

[28] Sewu D.D., Tran H.N., Ohemeng-Boahen G., Woo S.H. (2020). Facile magnetic biochar production route with new goethite nanoparticle precursor. Sci. Total Environ..

[29] Bourikas K., Kordulis C., Lycourghiotis A. (2005). Differential potentiometric titration: Development of a methodology for determining the point of zero charge of metal (hydr)oxides by one titration curve. Environ. Sci. Technol..

[30] Álvarez-Merino M.A., Fontecha-Cámara M.A., López-Ramón M.V., Moreno-Castilla C. (2008). Temperature dependence of the point of zero charge of oxidized and non-oxidized activated carbons. Carbon.

[31] Kosmulski M. (2019). The pH dependent surface charging and points of zero charge. VIII. Update. Adv. Colloid Interface Sci..

[32] Li X., Shen Q., Zhang D., Mei X., Ran W., Xu Y., Yu G. (2013). Functional Groups Determine Biochar Properties (pH and EC) as Studied by Two-Dimensional (13)C NMR Correlation Spectroscopy. PLoS ONE.

[33] Mohan D., Pittman C.U., Bricka M., Smith F., Yancey B., Mohammad J., Steele P.H., Alexandre-Franco M.F., Gómez-Serrano V., Gong H. (2007). Sorption of arsenic, cadmium, and lead by chars produced from fast pyrolysis of wood and bark during bio-oil production. J. Colloid Interface Sci..

[34] Tseng R.-L., Wu F.-C. (2008). Inferring the favorable adsorption level and the concurrent multi-stage process with the Freundlich constant. J. Hazard. Mater..

[35] Zhang Z., Yan L., Yu H., Yan T., Li X. (2019). Adsorption of phosphate from aqueous solution by vegetable biochar/layered double oxides: Fast removal and mechanistic studies. Bioresour. Technol..

[36] Cheng X., Huang X., Wang X., Sun D. (2010). Influence of calcination on the adsorptive removal of phosphate by Zn–Al layered double hydroxides from excess sludge liquor. J. Hazard. Mater..

[37] Jung K.-W., Ahn K.-H. (2016). Fabrication of porosity-enhanced MgO/biochar for removal of phosphate from aqueous solution: Application of a novel combined electrochemical modification method. Bioresour. Technol..

